# Advanced biosensor technology for mycotoxin detection

**DOI:** 10.3389/fnut.2025.1596690

**Published:** 2025-10-28

**Authors:** Xinya Tong, Ning Chen, Mengzhi Wang, Xiaodong Guo

**Affiliations:** ^1^College of Animal Science and Technology, Yangzhou University, Yangzhou, China; ^2^Institute of Animal Science, Xinjiang Academy of Agriculture and Reclamation Science, Shihezi, China; ^3^Joint International Research Laboratory of Agriculture & Agri-Product Safety of MOE, Yangzhou University, Yangzhou, China

**Keywords:** mycotoxins, biosensors, aptamer, nanomaterials, food safety

## Abstract

The increasing incidence of food safety related to mycotoxin contamination highlights a significant global challenge. Traditional mycotoxin detection methods, such as enzyme-linked immunosorbent assay (ELISA) and chromatographic techniques (e.g., high-performance liquid chromatography, HPLC), are often limited by prolonged analysis time, inadequate sensitivity, high costs, and operational complexity, which impede their practical application. In contrast, biosensor technology, possesses interdisciplinary advancements, has emerged as a key area of research due to its inherent advantages, including high sensitivity, rapid response, and cost-effectiveness. This review provides a comprehensive summary of recent technological advancements in the development of highly sensitive biosensors for mycotoxin detection. Furthermore, we propose that future developments should strategically incorporate artificial intelligence (AI), sustainable nanomaterials, and other innovative approaches to enhance biosensor performance significantly and expand their practical applicability in ensuring food safety.

## 1 Introduction

Mycotoxins, toxic secondary metabolites produced by filamentous fungi, are low molecular weight compounds known for their chemical stability and resistance to high temperatures. These widespread contaminants pose a significant threat to global food security and public health. It is estimated that approximately 25% of the world’s cereal crops are contaminated with mycotoxins annually, with substantial impacts extending to numerous other agricultural commodities (1). Currently, over 300 mycotoxins have been identified, with common and highly regulated examples including aflatoxins (AFs), ochratoxin A (OTA), zearalenone (ZEN), T-2 toxin (a trichothecene), deoxynivalenol (DON), and fumonisins (FBs), as summarized in Table 1. To mitigate food safety risks associated with mycotoxins, various detection methodologies have been developed. Traditional techniques, such as ELISA, HPLC, and polymerase chain reaction (PCR), continue to be widely utilized (2, 3). However, these methods have significant limitations, including long analysis time, inadequate sensitivity in certain contexts, variable specificity, reliance on expensive instrumentation, complex sample preparation procedures, and high operational costs (4). Consequently, there is an urgent need for the development of rapid, cost-effective, high specific, and sensitive detection technologies.

**Table 1:** Common mycotoxins in food safety.

Mycotoxin	Sources	Matrix	Health hazard
Aflatoxin	*Aspergillus flavus*	Peanuts, corn, rice	Chronic poisoning, growth disorders, carcinogenesis
Zearalenone	*Fusarium* spp. (*Fusarium graminis* and *Fusarium trilineatus*)	Corn, wheat, rice, barley, millet, oats	Increased estrogen levels, acute and chronic poisoning
Ochratoxin	*Aspergillus*	Wheat, corn, barley, oats, rye, rice, broomcorn	Liver disease, digestive dysfunction
Fumonisin	*Fusarium moniliforme*	Corn, corn products	The brain showed signs of leukomalacia
Vomitoxin	*Trichothecene*	Grain (used as feed, etc.)	Weakness, dizziness, diarrhoea and vomiting
T-2	*Fusarium*	Wheat, barley, corn, etc.	Anorexia, vomiting, diarrhea

**Figure 1:**
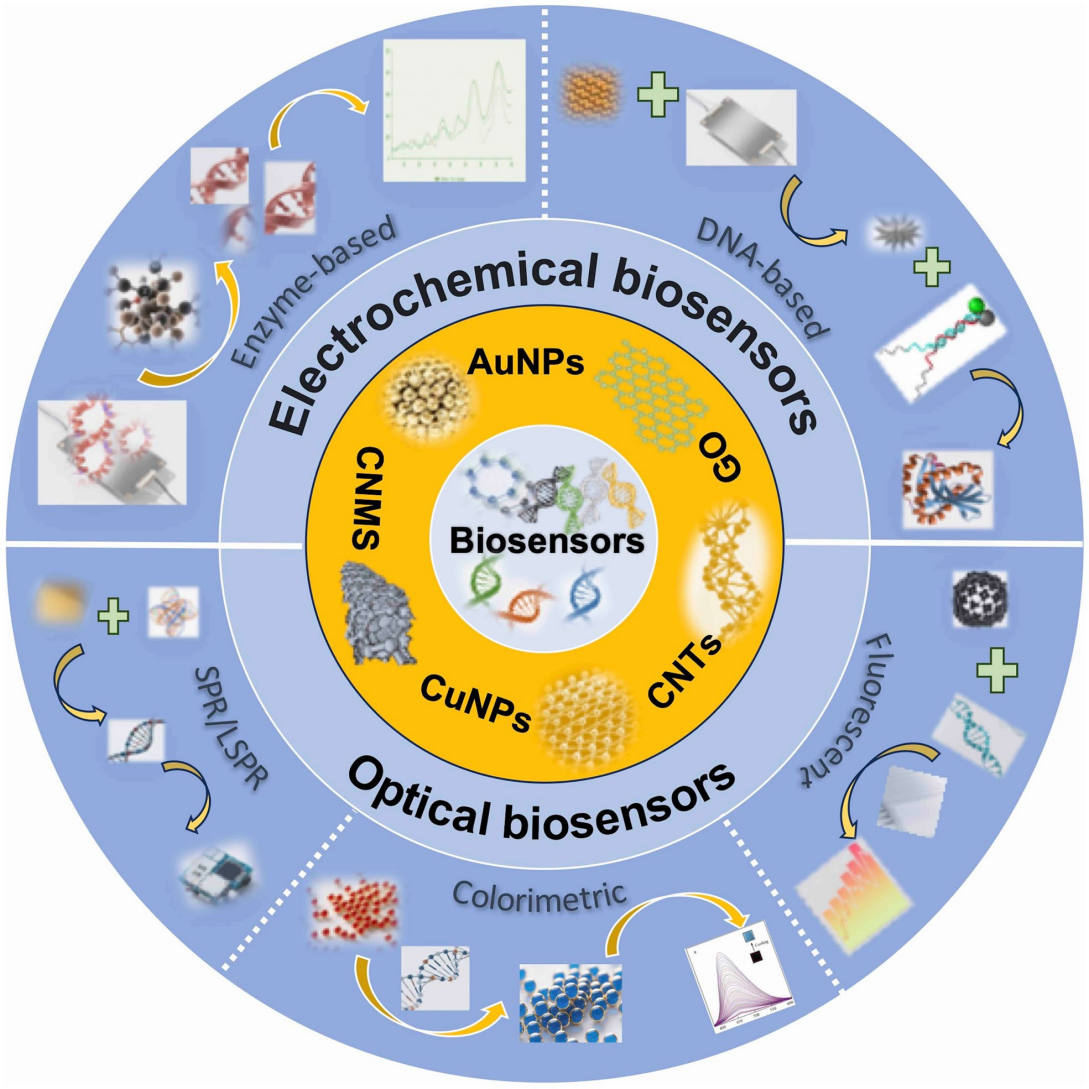
An overview of advanced biosensors and their working mechanism.

Biosensors, which are analytical devices that integrate a biological recognition element with a physicochemical transducer, present a promising solution to these challenges. Upon the specific interaction between the biorecognition element (such as an antibody, enzyme, aptamer, or nucleic acid) and the target mycotoxin, a physicochemical change occurs. This change is then converted by the transducer into a quantifiable signal (such as electrical, optical, piezoelectric, or thermal signal), which is subsequently processed and amplified for the qualitative or quantitative detection of the analyte, as illustrated in Figure 1 (5, 6). As inherently multidisciplinary tools, biosensors are utilized across various fields, including medicine, pharmaceuticals, and environmental monitoring (7). Advanced biosensor technologies for mycotoxin detection present significant advantages over traditional methods. While chromatographic techniques, such as HPLC and liquid chromatography-mass spectrometry (LC-MS), offer high sensitivity and specificity, they require expensive equipment, extensive sample preparation, and specialized personnel, making them impractical for rapid, on-site screening. Although immunoassays like ELISA are commercially available, they may encounter challenges such as false positives, reagent instability, and limited reproducibility.

In contrast, next-generation biosensors often utilize robust biorecognition elements such as aptamers (selected through Systematic Evolution of Ligands by Exponential Enrichment, SELEX), enzymes, or DNAzymes. The binding of the target triggers a molecular recognition event, which is transduced into measurable electrochemical or optical signals. Key advantages of these biosensors include high sensitivity, operational simplicity, rapid analysis time, and the potential for continuous monitoring. Sensitivity and specificity are further enhanced through signal amplification strategies (e.g., hybridization chain reaction-HCR, CRISPR/Cas systems) and the integration of novel nanomaterials (e.g., mesoporous silica nanoparticles, Au@Ag core–shell nanoparticles), which improve detection efficiency, stability, and transducer performance. Overall, these advancements address critical limitations of traditional methods by simplifying procedures, significantly reducing analysis time, and enabling adaptable on-site detection, thereby providing efficient and reliable solutions for mycotoxin monitoring in food safety (8, 9). This review explores the fundamental mechanisms and various applications of biosensors in mycotoxin detection, aiming to provide a theoretical foundation for future research and the development of more advanced biosensing platforms.

## 2 Recognition elements of biosensors

Current biosensor recognition elements primarily include enzymes, antibodies, and DNA-based probes. Each class exhibits distinct biochemical properties that define their applicability in mycotoxin detection.

### 2.1 DNA

DNA functions as a versatile biorecognition element in biosensors, utilizing its programmable structure, chemical stability, and molecular recognition specificity. These properties allow DNA probes to selectively hybridize with target analytes or complementary sequences, establishing precise detection methodologies. Advances in functional DNA structures (e.g., aptamers, DNAzymes) combined with nucleic acid amplification strategies have enabled significant applications in food safety monitoring, including mycotoxin detection, and clinical diagnostics such as tumor biomarker screening (10, 11). Key advantages include the structural predictability of double-stranded DNA ensuring consistent signal generation, inherent molecular recognition fidelity for ultralow concentration detection (12, 13), and adaptable target recognition extending to proteins, small molecules, and metal ions. DNA demonstrates superior thermal stability and batch to batch consistency relative to protein-based receptors.

Despite these advantages, DNA based recognition systems face significant limitations. Performance requires strict control of hybridization conditions as variations in temperature, pH, or ionic strength reduce binding efficiency (14). Developing high affinity probes demands labor intensive selection processes like SELEX and complex sequence engineering. Additionally, susceptibility to nuclease degradation necessitates stabilization strategies, a vulnerability not observed in protease resistant engineered antibodies.

### 2.2 Enzyme

Enzymes, as highly efficient biological catalysts, exhibit exceptional substrate specificity and catalytic activity essential for regulating fundamental physiological processes including cellular growth, differentiation, and apoptosis (15, 16). In biosensing systems, enzymes function as recognition elements by selectively binding target analytes and catalyzing biochemical reactions that generate detectable signals. Diverse enzyme classes, including oxidases (e.g., glucose oxidase), hydrolases (e.g., urease), and peroxidases, have been engineered for analytical applications in environmental monitoring, clinical diagnostics (e.g., renal urea quantification), and food/pharmaceutical quality control. The integration of enzymes with advanced nanomaterials (e.g., gold nanoparticles, graphene oxide) further enhances biosensor performance through amplified optical, electrical, and electrochemical signal transduction (17).

Key advantages of enzyme-based biosensors include their intrinsic catalytic amplification and application versatility. Enzymatic signal amplification mechanisms, such as peroxidase-catalyzed redox reactions, enable direct target detection without nucleic acid amplification steps, significantly improving sensitivity and operational simplicity. Substrate specificity further allows customization for diverse sensing platforms. However, limitations encompass stringent operational requirements and implementation costs. Similar to nucleic acids, enzymes require strict environmental control, particularly precise temperature regulation to maintain catalytic activity (18). Additionally, enzyme production and purification contribute to higher costs compared to synthetic recognition elements, though emerging technologies like wearable non-enzymatic electrochemical sensors offer cost-effective alternatives (19).

### 2.3 Antibody

Antibodies, immunoglobulins exhibiting high specificity for binding target antigens, constitute a primary class of natural biorecognition molecules (20). Their mechanism involves recognizing distinct antigenic epitopes, enabling rapid and precise identification of target analytes within complex biological matrices. Similar to enzymes, antibodies are characterized by high efficiency, specificity, and sensitivity. These intrinsic properties facilitate their integration with diverse functional materials, such as nanoparticles and graphene, leading to significant advancements in the detection of various proteins and receptors (21).

The utility of antibodies as biorecognition elements in biosensors is underpinned by several key advantages: (i) The specific antigen-antibody interaction represents a fundamental biological recognition process, exploited in numerous therapeutic and diagnostic applications. (ii) Their exceptional sensitivity and specificity render them highly effective tools, particularly in demanding fields like cancer diagnostics and targeted therapies. However, the application of antibodies in biosensing is accompanied by notable limitations: (i) Antibody production is typically a resource-intensive process, requiring significant time, materials, and incurring high costs. (ii) Batch-to-batch variability can lead to inconsistencies in sensitivity and specificity, impacting assay reproducibility. Consequently, addressing the inherent complexity and ensuring the stability of antibodies are critical considerations during the development and fabrication of antibody-based biosensors (22).

## 3 Classification and application of biosensors

Biosensors integrate biological recognition elements (enzymes, antibodies, cells) with sensing technology to detect specific analytes and monitor biological processes (9). Typically, biosensors comprise three essential components: a biological recognition element, a transducer, and a signal processing system (23). For mycotoxin detection, optical, electrochemical, and photoelectrochemical biosensors represent the primary modalities, categorized according to transduction principles (24), with each platform demonstrating distinct advantages in sensitivity, selectivity, and operational efficiency (25). Furthermore, nanomaterials enhance detection performance through their exceptional physicochemical properties and high surface area-to-volume ratios, improving bioreceptor immobilization and signal amplification in nanomaterial-based biosensors for mycotoxin analysis (26).

### 3.1 Electrochemical biosensors

Electrochemical biosensors represent an important class of analytical devices that utilize electrochemical transduction to convert specific bio-recognition events into quantifiable electrical signals. This integration of biorecognition elements (e.g., enzymes, antibodies, nucleic acids) with electrochemical transducers offers significant advantages, including high sensitivity, precision, and robustness (27). Compared to alternative biosensing platforms, electrochemical biosensors are often characterized by simpler instrumentation, faster response time, and reduced requirements for sample pretreatment (28). A key strength lies in their ability to amplify minute molecular interactions into readily measurable electrical outputs. Furthermore, electrochemical technology facilitates straightforward miniaturization and integration with portable systems such as microfluidic chips and smartphones, enhancing their suitability for point-of-care testing (POCT) across diverse environments. In resource-limited settings lacking sophisticated laboratory infrastructure, electrochemical biosensors thus represent a highly viable option for rapid and reliable analyte detection. Consequently, their distinct advantages have driven widespread adoption in critical fields including food safety assurance, environmental monitoring, clinical diagnostics, and biosecurity (29).

#### 3.1.1 Classification of electrochemical biosensors

Electrochemical biosensors can be categorized based on several criteria. Classification by electrochemical detection technique yields categories such as amperometry, voltammetry (including cyclic, differential pulse, and square wave variants), electrochemical impedance spectroscopy (EIS), and potentiometry (30). Alternatively, categorization based on electrode material and modification strategies includes groups like metal electrode-based (e.g., Au, Pt), carbon-based (e.g., glassy carbon, screen-printed carbon, graphene), and modified electrode-based biosensors (e.g., with nanomaterials, polymers) (31). Among the diverse configurations, enzyme-based, DNA-based, and nanomaterial-enhanced platforms are particularly prevalent and will be discussed in detail below.

##### 3.1.1.1 Enzyme-based electrochemical biosensors

Enzyme-based electrochemical biosensors exploit the high catalytic activity and substrate specificity of enzymes as biorecognition elements. This enables the development of sensors with excellent selectivity, inherent signal amplification (via catalytic turnover), relatively simple design, and cost-effectiveness, often obviating the need for complex separation steps (32). Nevertheless, achieving efficient enzyme immobilization on transducer interfaces without compromising bioactivity constitutes a persistent challenge. Current immobilization strategies exhibit limitations that may adversely affect sensor performance metrics and reproducibility (33). Moreover, the intrinsic environmental sensitivity of enzymes poses a significant limitation. Besides, their catalytic activity is readily compromised by factors such as temperature extremes, pH variations, and humidity (34). Consequently, maintaining enzyme stability and activity under operational conditions represents a major research focus for advancing this biosensor class.

##### 3.1.1.2 DNA-based electrochemical biosensors

DNA-based electrochemical biosensors utilize the high specificity of Watson-Crick base pairing for target recognition. Typically, a single-stranded DNA (ssDNA) probe, complementary to the target sequence (e.g., DNA, RNA, or specific molecules recognized by aptamers), is immobilized on an electrode surface (e.g., gold or carbon). Hybridization with the target analyte induces measurable changes in interfacial properties (e.g., charge transfer resistance, capacitance, or faradaic current), which are transduced into an electrical signal (35). Beyond conventional hybridization sensors, functional DNA molecules, such as aptamers (with high affinity for specific non-nucleic acid targets like proteins or small molecules) and DNAzymes (catalytic DNA sequences), are increasingly employed. Aptamers provide high selectivity comparable to antibodies, while DNAzymes offer catalytic signal amplification analogous to enzymes, significantly enhancing detection sensitivity (36, 37).

##### 3.1.1.3 Electrochemical nanobiosensors

Electrochemical nanobiosensors incorporate nanomaterials (e.g., metal nanoparticles like Au and Ag, carbon nanotubes, graphene, quantum dots) to significantly enhance performance. These nanomaterials function by increasing the effective electrode surface area for bioreceptor immobilization, improving electron transfer kinetics, and sometimes providing intrinsic catalytic properties or labels (38). This synergy between nanomaterials and biorecognition elements (exploiting specific interactions like antibody-antigen binding, enzyme-substrate reactions, or nucleic acid hybridization) leads to substantially improved sensitivity, selectivity, and overall sensor performance. Consequently, electrochemical nanobiosensors exhibit broad applications, particularly in the highly sensitive detection of trace analytes such as disease biomarkers, environmental pollutants, and heavy metals.

#### 3.1.2 Emerging application trends of electrochemical biosensors

Electrochemical biosensors are undergoing rapid evolution, driven by the persistent demand for sensitive, selective, and user-friendly analytical tools, particularly within critical areas like mycotoxin monitoring for food safety. Recent advancements prominently feature strategic innovations in nanomaterials and sophisticated signal amplification strategies, although significant challenges remain to be addressed.

A prominent trend in electrochemical mycotoxin sensing involves the strategic design of advanced nanocomposites to significantly enhance analytical performance. For instance, Huang et al. (39) developed a Bi_2_S_3_-embedded carbon nanofiber (Bi_2_S_3_@CNF) nanocomposite as a sensing platform for ZEN detection. This Bi_2_S_3_@CNF hybrid combines advantageous properties where Bi_2_S_3_ contributes electrocatalytic activity while the CNF matrix provides conductivity and stability. When immobilized on a glassy carbon electrode (GCE), the Bi_2_S_3_@CNF hybrid demonstrated enhanced electron transfer kinetics, broad linear detection range from 0.125 to 1951 μM, and low limit of detection (LOD) of 0.61 μM. These improvements are attributed to the optimized interfacial properties and the abundance of active sites that facilitate efficient ZEN oxidation. Similarly, Hui et al. (40) employed gold nanoparticles (AuNPs) functionalized with horseradish peroxidase (HRP) and thiolated DNA to construct a DNA-AuNPs-HRP nanoprobe for AFB1 detection. The AuNPs served as a high-surface-area scaffold for HRP immobilization and DNA conjugation, while HRP catalyzed the hydroquinone/H_2_O_2_ redox reaction, amplifying the electrochemical signal. Coupled with exonuclease I-assisted target recycling, this dual-amplification strategy achieved exceptional sensitivity detecting concentrations as low as 3.3 × 10^−4^ ng/mL. To accelerate nanomaterial innovation, Zhang et al. (41) further utilized Ti_3_C_2_ MXene nanosheets decorated with *in-situ*-grown gold nanorods (AuNRs) and electrostatically loaded Ru(bpy)_3_^2+^ (Ti_3_C_2_@AuNRs-Ru) for electrochemiluminescence (ECL) aptasensing of T-2 toxin. The Ti_3_C_2_ MXene contributed high conductivity and catalytic activity, while AuNRs enabled stable aptamer conjugation via Au–S bonds. The resulting nanocomposite enhanced ECL efficiency, enabling detection of T-2 toxin down to 6.44 fg/mL. In parallel, Akpınar et al. (42) contributed to this innovation trend through a simplified effective nanomaterial approach. They synthesized silicon dioxide nanoparticles (SiNPs) to modify disposable screen-printed electrodes (SPEs) for electrochemical detection of patulin–DNA interactions. The SiNPs increased the electrode surface area by 2.3-fold and amplified signals by 2-fold, enabling patulin detection at 1.15 μg/mL within a linear range of 3.2–20 μg/mL. While less sensitive than multilayer composites (e.g., Ti_3_C_2_@AuNRs-Ru), the SiNP-SPE system offers advantages in cost, disposability, and direct biomolecular interaction analysis. These nanocomposites demonstrate how tailored material interfaces address diverse requirements in mycotoxin monitoring.

**Figure 2:**
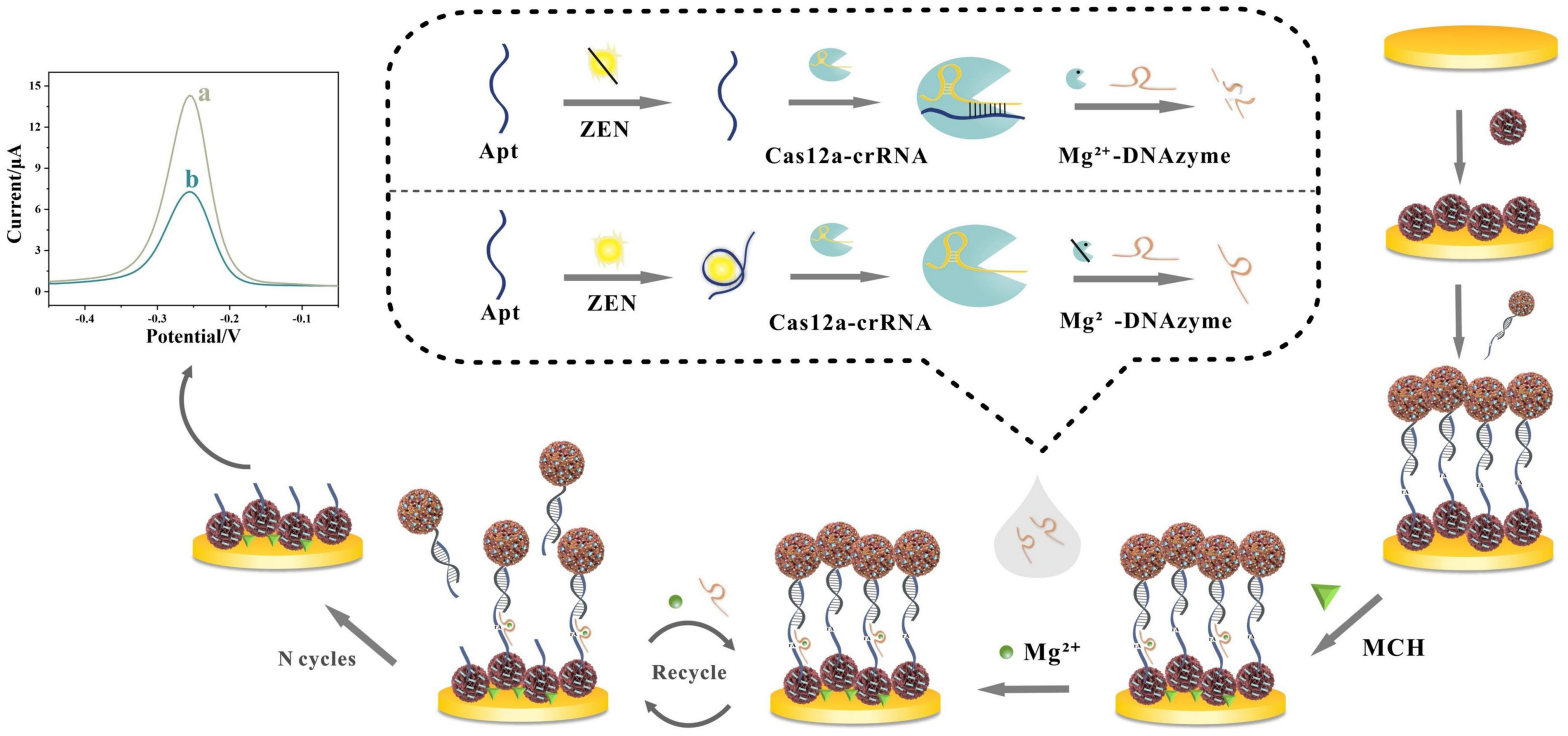
Innovative design of electrochemical aptasensor against ZEN detection by the combination of CRISPR/Cas12a-mediated and DNAzyme-assisted cascade dual-enzyme transformation strategy. Reproduced from Yan et al. (43) with permission from Elsevier.

Beyond nanomaterial engineering, sophisticated biological and enzyme-free amplification mechanisms are increasingly integrated to boost sensitivity. As shown in Figure 2, Yan et al. (43) developed a CRISPR/Cas12a-activated cascade for ultra-trace ZEN detection, where target-bound aptamer triggered Cas12a trans-cleavage to degrade Mg^2+^-dependent DNAzyme probes. This initiated DNAzyme-assisted catalytic recycling on electrode-bound PtPd@Fe_3_O_4_-MB labels, amplifying current reduction. Combined with conductive NH_2_-MnO_2_/Pd@Au nanorod composites for signal enhancement, this dual-enzyme transformation achieved an unprecedented LOD of 6.27 × 10^−6^ ng/mL and 93.89–107.33% recovery in corn flour, eliminating thermocycling steps. Similarly, Yu et al. (44) developed an enzyme-free AFB1 aptasensor using HCR amplification. Target-induced DNA release triggered autonomous assembly of THI/Au@PtNP-labeled hairpins on AuNPs/Co-MOF electrodes. This HCR-driven assembly utilizes Co-MOF’s porosity for high-density DNA anchoring and Au@PtNP’s catalytic activity for thionine signal amplification, achieving an LOD of 0.012 pg/mL while maintaining specificity against structural analogs.

Simultaneously, significant efforts have been directed towards reducing cost and operational complexity to enhance practical utility. Wang et al. (45) designed a label-free electrochemical aptasensor based on MXene (Ti_3_C_2_Tx) nanosheets for AFB1 detection, circumventing the high cost and complexity of chromatographic methods. This sensor quantified AFB1 by monitoring impedance changes via EIS, where AFB1-aptamer binding hindered electron transfer. The platform demonstrated good recovery (97.8–111.52%) and potential for multiplexing via aptamer exchange.

Parallel advances focus on biosensors capable of simultaneous detection of multiple mycotoxins, representing a critical advancement for comprehensive food safety monitoring. Li et al. (46) engineered an electrochemical biosensor using DNA tetrahedral nanoscaffolds (TDNs) for simultaneous quantification of AFB1 and OTA. This platform operated through toxin-specific binding, inducing structural dissociation of TDNs that modulates electrode surface currents. Differential pulse voltammetry (DPV) resolved discrete signals for both toxins without cross-interference, achieving ultra-sensitive LODs of 3.5 pg/mL for AFB1 and 2.4 pg/mL for OTA. The biosensor demonstrated high-fidelity performance in complex peanut matrices, with linear ranges of 0.05–360 ng/mL for AFB1 and 0.05–420 ng/mL for OTA. Complementing this approach, Wang et al. (47) developed a multiplex MXene-based aptasensor integrated with a portable multi-channel electrochemical system for simultaneous detection of AFB1, OTA, and ZEN in corn. The sensor incorporated a tripartite electrode array functionalized with toxin-specific aptamers, exploiting MXene’s high electrical conductivity and substantial specific surface area to augment signal transduction efficiency. This configuration demonstrated a 3.5-fold efficiency improvement over single-analyte platforms, reaching unprecedented detection sensitivity of 41.2 pg/mL for AFB1, 27.6 pg/mL for OTA, and 33.0 pg/mL for ZEN alongside robust anti-interference capabilities.

In summary, these emerging trends demonstrate collaborative progress in pushing the frontiers of mycotoxin detection via electrochemical biosensing. The key driving factors include nanomaterial engineering, high-efficiency signal amplification cascades, and pragmatic approaches emphasizing affordability and operational simplicity.

#### 3.1.3 Challenges of electrochemical biosensors

Despite significant advances in materials and signal amplification, electrochemical biosensors continue to face challenges that hinder their real-world application for mycotoxin detection. Achieving sufficient selectivity remains a critical barrier, as electrochemical signals are vulnerable to matrix interference from components such as food ions and non-specific binding events. These factors increase the risk of generating false positives or negatives. Addressing this issue requires the development of higher-affinity bioreceptors such as engineered aptamers and the integration of effective anti-fouling mechanisms. Stability concerns present another major obstacle. Bioreceptors, including enzymes and antibodies, degrade under operational stresses such as fluctuations in pH or temperature, while nanomaterials like MXenes are prone to oxidation, which limits their practical use. Implementing robust immobilization techniques and creating degradation-resistant interfaces are essential solutions.

A further challenge involves balancing sensitivity gains with operational complexity. Sophisticated amplification strategies such as CRISPR systems and enzyme cascades increase cost and procedural demands, conflicting with point-of-need application requirements. Simplifying these architectures while maintaining high performance is crucial. Additionally, demonstrating reliable performance in real samples is exceptionally difficult. Matrix effects in unprocessed food or biological fluids can significantly alter sensor responses, necessitating extensive validation against gold-standard methods such as HPLC or LC-MS, as well as consistent demonstration of high recovery rates across diverse sample types. Successfully addressing these interconnected challenges related to selectivity, stability, complexity management, and real-sample reliability is fundamental for translating electrochemical biosensors from research laboratories to practical field implementation.

### 3.2 Optical biosensors

Optical biosensors represent a class of advanced analytical devices that utilize principles of light-matter interaction (e.g., refraction, reflection, absorption) integrated with biological recognition elements. As third-generation biosensing platforms, they transduce biorecognition events into quantifiable optical signals through specialized transducers (48). Based on the underlying transduction mechanism, optical biosensors are commonly categorized into types such as surface plasmon resonance (SPR), localized surface plasmon resonance (LSPR), refractometric, and interferometric sensors. These platforms offer significant advantages, including relative intuitiveness, rapid response times, and potential for portability (49). Consequently, these attributes have established optical biosensors as critical tools in food, environmental pollutant monitoring, and integration with smartphone-based sensing technologies.

#### 3.2.1 Classification of optical biosensors

The growing concern over mycotoxin contamination and its impact on food safety in recent years has intensified the demand for detection methods that surpass traditional techniques in terms of convenience and speed. Optical biosensors, owing to their superior performance characteristics, have emerged as promising solutions to meet this demand.

##### 3.2.1.1 Resonance-based biosensors

Plasmon resonance-based biosensors are among the most prevalent label-free detection technologies. They exploit resonance phenomena to monitor interactions between analytes and biorecognition elements, providing real-time molecular information. A typical system comprises three core components: an optical reader, a biorecognition element, and a sampling unit. This category primarily includes SPR and LSPR biosensors. In fact, SPR biosensors operate by detecting changes in the refractive index of the medium adjacent to a thin metal film (commonly gold) upon biomolecular binding. They are characterized by high specificity, sensitivity, and relatively low operational costs (50). Conversely, LSPR biosensors utilize confined plasmonic oscillations within metallic nanostructures such as gold nanoparticles. This mechanism enables miniaturized sensor designs while enhancing spatial resolution. Significant performance improvements in both SPR and LSPR platforms have been attained through nanomaterial engineering, including the development of nanostructured surfaces, graphene interfaces, and optimized metallic architectures. These innovations collectively increase sensitivity and lower detection limits (51). Such capabilities establish plasmonic biosensors as indispensable tools for quantitative analysis in complex matrices, from food contaminants to clinical biomarkers.

##### 3.2.1.2 Colorimetric biosensors

Colorimetric biosensors provide a direct, convenient, and rapid visual detection modality. Their operation relies on the generation of a distinct, measurable color change upon specific binding between the analyte and the recognition element, enabling quantitative or semi-quantitative analysis (52). AuNPs are extensively utilized in fabricating colorimetric biosensors due to their strong distance-dependent optical properties, ease of synthesis, functionalization versatility, and high stability (53). The visible color shift (e.g., red to blue for AuNP aggregation) facilitates straightforward interpretation, often without sophisticated instrumentation.

##### 3.2.1.3 Fluorescent biosensors

Fluorescent biosensors detect and quantify target analytes based on measurable changes in fluorescence properties (e.g., intensity, wavelength shift, lifetime, anisotropy) induced by the specific binding event. They represent an extremely versatile and widely used sensor class, offering both visual (qualitative) and real-time quantitative capabilities (54). The characteristic change typically originates from the fluorophore itself (e.g., fluorescent dye, quantum dot) or its microenvironment upon interaction with the recognition molecule. Detection is accomplished using devices ranging from microscopes to dedicated fluorimeters or plate readers. Recent scientific developments focus on applying fluorescent biosensors to food safety monitoring. Integration with nanomaterials can significantly amplify fluorescence signals, while the development of dual-mode systems combining colorimetric and fluorescence detection has further enhanced their utility and reliability in this domain (55).

##### 3.2.1.4 Optical nanobiosensors

Optical nanobiosensors constitute a refined class of optical sensing platforms that operate through specific binding interactions between target biomolecules and complementary recognition elements immobilized on nanomaterial-functionalized transducer interfaces (56). This specific binding event induces measurable alterations in the optical properties at the sensor interface, detectable through changes in parameters such as fluorescence intensity, absorption spectrum, scattering profile, or refractive index. The integration of nanomaterials imparts critical advantages distinct from conventional optical sensors. Notably, these nanomaterials exhibit unique optical properties, including intense surface plasmon resonance effects, high fluorescence quantum yields, and large scattering cross-sections, which significantly enhance the interaction between biomolecules and the sensor surface. This leads to amplified optical signal intensity and improved stability. Furthermore, the surface chemistry of nanomaterials can be precisely engineered to achieve highly selective recognition of specific biomolecular targets (57). Moreover, nanomaterials facilitate efficient and stable immobilization of biorecognition elements through diverse strategies such as physical adsorption, chemical covalent bonding, and bio-specific interactions. This capability enables controlled orientation of the biomolecules on the sensor surface, thereby optimizing binding efficiency and interface stability. The practical impact of these advantages is evident in specific sensor types. For instance, quantum dot-based fluorescent sensors enable highly sensitive biomolecule detection, while LSPR biosensors utilizing gold nanoparticles excel in monitoring biomolecular interactions (58). Consequently, optical nanobiosensors offer enhanced performance crucial for demanding applications.

#### 3.2.2 Emerging application trends in optical biosensors

Optical biosensors are increasingly overcoming limitations inherent in conventional detection methodologies, such as time-consuming procedures, operational complexity, and restricted accuracy. This advancement is driven by strategic integration with nanotechnology, microfluidics, two-dimensional materials, and chip-based systems. These cross-disciplinary synergies significantly enhance detection sensitivity, accuracy, and rapidity, facilitating deployment across diverse analytical scenarios from environmental monitoring to POC diagnostics.

Early innovations demonstrate the power of simplifying detection workflows. Shahdeo et al. (59) developed a microfluidic paper-based analytical device (μPAD) for rapid on-site detection of OTA in corn and groundnut. Their approach employed a 36-mer aptamer coupled with gold nanoparticles (AuNPs) in a colorimetric assay. Fabricated by patterning hydrophobic barriers on filter paper, the μPAD featured distinct control, detection, and sample collection areas. Target binding in the detection zone triggered aptamer displacement from AuNPs, causing salt-induced nanoparticle aggregation and a visible color shift from red to gray. This method achieved an LOD of 545.45 ng/mL in corn and 95.69 ng/mL in groundnut within 5 min, eliminating sample extraction or cleanup. Validation against HPLC confirmed practicality despite higher detection limits, as the μPAD bypassed lengthy extraction steps and reduced recovery variability. Its operational simplicity and cost-effectiveness contribute it as a viable tool for preliminary field screening in resource-limited agricultural settings.

A significant leap in biosensing capability arises from integrating CRISPR-Cas systems with nanomaterials. Wang et al. (60) engineered a CRISPR/Cas12a-driven ratiometric fluorescent aptasensor for OTA detection. This system combined HCR amplification with HRP-induced inner-filter effect. OTA recognition through competitive aptamer binding triggered Cas12a trans-cleavage, suppressing HCR assembly on magnetic beads. This reduced HRP loading and consequently limited the conversion of o-phenylenediamine to fluorescent 2,3-diaminophenazine, while preserving the emission of 2-amino terephthalic acid. The resulting fluorescence ratio signal achieved exceptional sensitivity with an LOD of 0.0417 pM across 0.1 pM to 10 nM dynamic range and recoveries of 90.1–110.6% in complex matrices. Crucially, this method circumvents target pre-amplification and mitigates environmental interference through intrinsic self-calibration, marking a notable advancement for non-nucleic acid targets. Furthermore, to enhance CRISPR-based detection, Liu et al. (61) developed a colorimetric biosensor utilizing Fe-N-C single-atom nanozymes (SAzymes) for AFB1 detection. Exploiting the peroxidase-like activity of Fe-N-C SAzymes, the system catalyzed the oxidation of TMB to generate a visible color change, achieving an ultralow LOD of 1.5 × 10^−4^ ng/mL and significantly below regulatory limits. Fe-Co magnetic nanoparticles enabled magnetic separation, reducing background noise and cost. This work elucidated the catalytic mechanism of Fe-N_4_ sites and established a generalizable platform adaptable to other non-nucleic acid targets via aptamer substitution.

**Figure 3:**
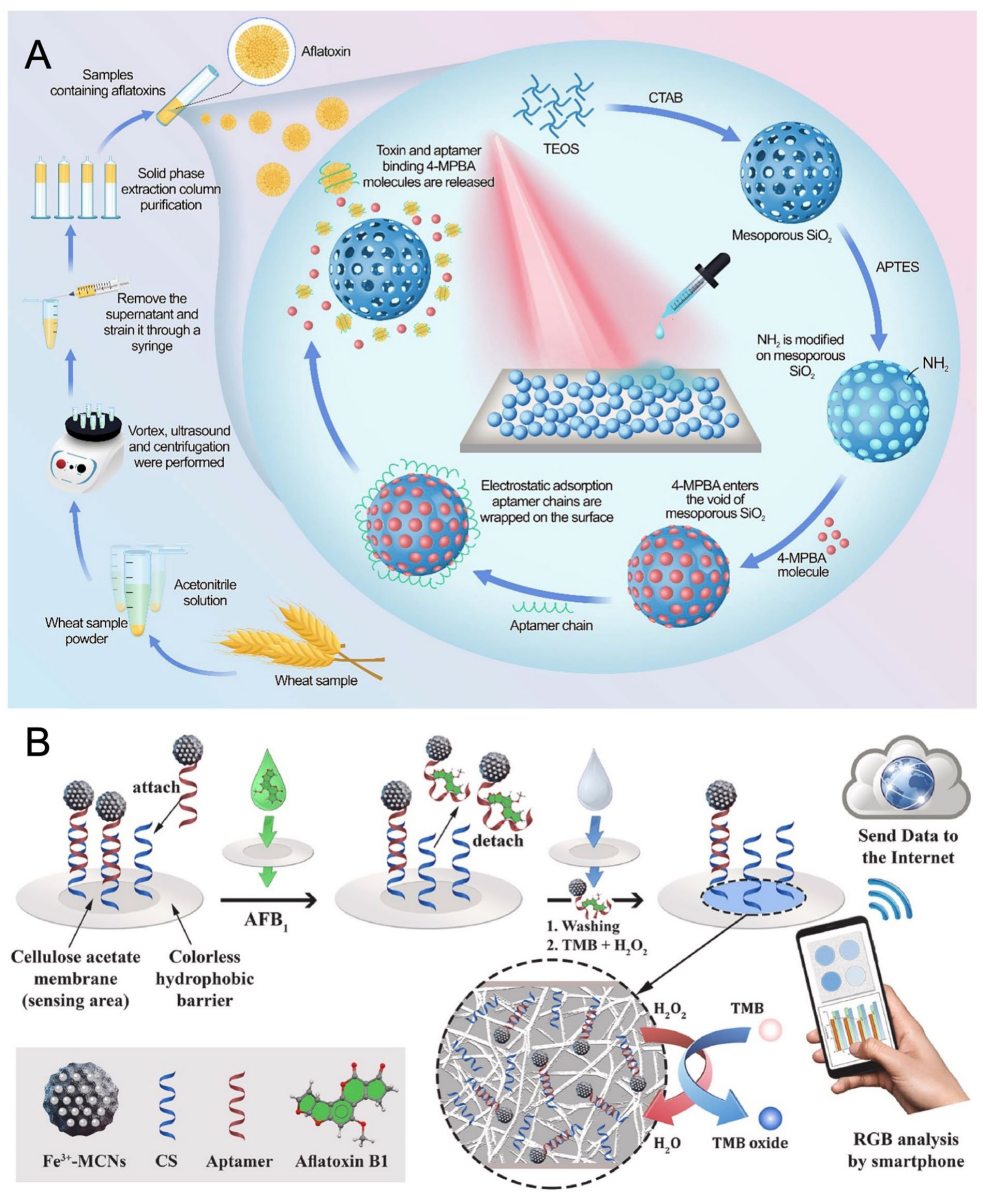
**(A)** Mechanism illustration of SERS-based aptasensor toward AFB1 detection based on AFB1-responsive mesoporous silica nanoparticles. Reproduced from Jiao et al. (63) with permission from Elsevier. **(B)** Working principle of colorimetric platform for determination of AFB1 by using field-deployable sensing with an integrated paper-based biosensor. Reproduced from Moshirian-Farahi et al. (65) with permission from Elsevier.

Addressing challenges in complex matrices, Zhang et al. (62) synthesized core–shell Au@PB@Au nanoparticles embedded with Prussian blue (PB) for surface-enhanced Raman spectroscopy (SERS). PB emits in the Raman-silent region (1,800–2,300 cm^−1^), minimizing background interference and enabling self-calibration. Coupled with CRISPR/Cas12a, the platform converted AFB1 detection into nucleic acid signals via exonuclease-assisted amplification, achieving an LOD of 3.55 pg/mL. Validated in milk and soy sauce with recoveries of 94–116% and low RSDs, it outperformed fluorescence and electrochemical techniques in sensitivity. Extending SERS-based approach, Jiao et al. (63) engineered an aptamer-gated biosensor utilizing mesoporous silica nanoparticles (MSNs) for AFB1 quantification. The functionalized MSNs encapsulated the reporter molecule 4-mercaptophenylboronic acid (4-MPBA), with surface confinement mediated by AFB1-specific aptamers. As illustrated in Figure 3A, Target binding induced aptamer detachment, releasing 4-MPBA which generated a concentration-dependent SERS signal upon capture by Au@Ag nanoparticles. This yielded a linear range of 0.1–5 ng/mL and an LOD of 0.03 ng/mL, with assessment in wheat showing statistical parity to HPLC-FLD and detection within 30 min using portable Raman equipment.

Portability and multiplexing represent key frontiers. Based on Jia et al. (64), a portable chemiluminescence optical fiber aptasensor system was engineered for ultrasensitive multiplex mycotoxin detection. In this competitive design, mycotoxin binding limits aptamer attachment to an SSB-functionalized fiber. Subsequent introduction of SA-Bio-HRP and chemiluminescent substrate generates a signal inversely proportional to mycotoxin levels. The system achieved remarkably low LODs of 0.032 pg/mL for AFB1, 0.015 pg/mL for FB1, 0.423 pg/mL for OTA, and 0.275 pg/mL for ZEN, respectively. It demonstrated reliable multiplexed quantification in complex food matrices with minimal cross-reactivity. Moshirian-Farahi et al. (65) further advanced field-deployable sensing with an integrated paper-based biosensor. As shown in Figure 3B, it incorporated Fe^3+^-doped mesoporous carbon nanospheres (Fe^3+^-MCNs) as peroxidase-mimetic nanozymes for AFB1 detection. Target binding released aptamer-functionalized nanozymes hybridized to complementary strands on cellulose acetate nanofibers. Spatial confinement within the nanofiber network intensified the colorimetric output sevenfold by preventing signal dilution, coupled with the high catalytic efficiency of Fe^3+^-MCNs. Excitingly, this design achieved an ultralow LOD of 3.9 pg/mL, with smartphone-based RGB analysis enabling quantitative on-site detection in 3 min.

Parallel innovations specifically target multiplexed detection using nanomaterial properties. Cai et al. (66) constructed a fluorescent aptasensor using functionalized graphene oxide (FGO) and Förster resonance energy transfer (FRET) for simultaneous quantification of AFB1 and AFM1. Cy3 and Cy5-labeled aptamers adsorb onto FGO, quenching fluorescence via FRET effect. Target binding induces desorption and fluorescence recovery, enabling dual-channel detection with LODs of 8.7 pg/mL for AFB1 and 20.1 pg/mL for AFM1, and further validated in peanuts and milk, respectively. Similarly, Wang et al. (67) developed a portable dual-color FRET aptasensor employing MoS_2_ nanosheets and carbon dots/CdZnTe quantum dots for simultaneous OTA and AFB1 detection. Fluorescently labeled aptamers adsorb onto MoS_2_ quenchers, and toxin binding triggers complex dissociation and fluorescence restoration. Optimization yielded LODs of 0.038 ng/mL for OTA and 0.041 ng/mL for AFB1, representing a significant sensitivity improvement together with minimal interference from other mycotoxins.

Wang et al. (68) demonstrated a representative advancement in multiplexed portability through biocompatible multicompartmental hydrogel microspheres (MCPMs) engineered using microfluidics. These spatially compartmentalized microspheres simultaneously capture patulin, AFB1, and OTA at single-particle level. Signal amplification via an HCR triggered by binding generates a single-fluorophore fluorescence signal readable with a portable imager, achieving low LODs of 0.033 ng/mL for patulin, 0.053 ng/mL for AFB1, and 0.10 ng/mL for OTA, positioning it as a promising POC testing platform. In contrast, Song et al. (69) pursued ultra-high sensitivity with a SERS aptasensor based on a gold nanoparticle-functionalized silica photonic crystal microsphere (SPCM) array. Capitalizing on the localized electromagnetic field enhancement intrinsic to SPCMs, competitive displacement of dye-loaded SERS nanotags by target mycotoxins at aptamer-conjugated interfaces generated quantifiable, concentration-dependent SERS signal attenuation. This strategy achieved exceptionally low LODs of 0.36 pg/mL for AFB1 and 0.034 pg/mL for OTA, along with wide linear ranges. The approach effectively utilizes the inherent multiplexing capability enabled by distinct Raman signatures, although it requires more sophisticated instrumentation compared to simpler POC platforms.

The trend towards sophisticated signal amplification is exemplified by Liu et al. (70) through a programmable entropy-driven dynamic system, implementing a fluorescent aptasensor for simultaneous quantification of AFB1 and OTA in herbal medicines. Mycotoxin binding triggers the release of a catalytic strand from a blocked complex, initiating a toehold-mediated strand displacement cascade that cyclically amplifies the signal. Using distinct fluorophore-quencher pairs (FAM/BHQ1 for AFB1, ROX/BHQ2 for OTA), the system achieved LODs of 5.7 pg/mL for AFB1 and 3.4 pg/mL for OTA, respectively. This enzyme-free, isothermal approach, validated in complex matrices, exemplifies a modular, cost-effective, and sensitive alternative to chromatographic techniques through nucleic acid programmability.

In summary, the evolution of optical biosensors for mycotoxin detection is propelled by strategic cross-disciplinary integration, overcoming key limitations of conventional methods. This convergence yields three dominant trends: achieving unprecedented sensitivity through novel amplification strategies and enhanced nanomaterials; advancing portability and field-deployment via paper-based devices, microfluidics, and smartphone coupling; and enabling robust multiplexed detection using nanomaterial-enabled FRET, spatial compartmentalization, and distinct optical signatures. Taken together, these innovations are forging highly sensitive, rapid, user-friendly, and cost-effective platforms poised to transform food safety monitoring and POC diagnostics. Future efforts will focus on workflow simplification, expanded multiplexing, and enhanced real-world application.

### 3.3 Dual/multi-mode biosensor applications

Conventional single-mode biosensors face inherent limitations in complex food matrices, where matrix interference and false positives compromise reliability. Dual/multi-mode platforms strategically overcome these constraints by integrating orthogonal signal transduction pathways. This innovation provides intrinsic cross-validation, significantly enhancing accuracy for critical mycotoxins like AFB1 and OTA while catalyzing design in sensor architecture. Representative case studies demonstrate distinct design principles and performance compromises inherent to optical, electrochemical, and hybrid sensing modalities.

Optical dual-mode systems predominantly prioritize extreme sensitivity or field adaptability. For instance, Wang et al. (71) developed a dual-mode aptasensor exploiting fluorescence and SERS for OTA quantification. The platform utilized gold nanostars functionalized with OTA-specific aptamers and gold nanospheres conjugated with Cy3-labeled complementary DNA, which self-assembled into satellite structures. This configuration inherently quenched fluorescence signals due to fluorophore proximity to gold surfaces while simultaneously generating intense SERS signals through plasmonic hot-spot formation at nanogap junctions. Target recognition event triggered structural dissociation, restoring fluorescence emission and diminishing SERS intensity proportionally to OTA concentration. The fluorescence mode achieved an LOD of 0.17 ng/mL across a linear dynamic range of 1–100 ng/mL, while the SERS mode demonstrated exceptional sensitivity with an LOD of 1.03 pg/mL within a linear working range of 5–250 pg/mL. Importantly, this orthogonal dual-signal approach not only spanned three orders of magnitude dynamically but also provided inherent mutual verification, substantially enhancing result reliability. In parallel, Mou et al. (72) developed a portable dual-mode aptasensor for OTA using a synthesized coumarin-benzothiazole probe (DRI) that selectively bound the G-quadruplex structure of an OTA-specific aptamer. This binding concurrently generated a strong fluorescence signal and induced a distinctive purple-to-blue color transition. Upon OTA addition, competitive displacement of DRI quenched fluorescence with an LOD of 0.01 μM and reversed the solution color from blue to purple with an LOD of 0.1 μM. Integration with a smartphone for RGB analysis enabled robust on-site visual screening, assessed in grape juice with excellent recoveries from 94.8 to 98.1%. Meanwhile, Wu et al. (73) developed a CRISPR/Cas12a-driven multimodal system with G-quadruplex DNAzyme (G4-DNAzyme) signal amplification for AFB1 detection. Target binding liberated cDNA to activate Cas12a, which subsequently cleaved and inactivated the G4-DNAzyme. This diminished the DNAzyme’s peroxidase-like activity, reducing the catalytic conversion of TMB to TMBox. The reduction was quantified by colorimetric absorbance change achieving an LOD of 0.85 pg/mL, via SERS intensity decrease of TMBox reaching an LOD of 0.79 pg/mL, and through fluorescence quenching with an LOD of 1.65 pg/mL. The colorimetric signal further facilitated practical smartphone-assisted visual analysis in food samples.

Building on optical foundations, electrochemical hybrids deliver essential robustness against matrix interference in complex samples through integrated transduction pathways. Zhang et al. (74) designed a microfluidic platform integrating electrochemical and colorimetric transduction for ultrasensitive AFB1 monitoring. Tetrahedral DNA nanostructures ensured precise probe orientation, while Au/Ni-Co layered double hydroxide nanocages served as highly efficient peroxidase-mimetic nanozymes. The EC mode exhibited a remarkably low LOD of 0.071 pg/mL spanning seven orders of magnitude from 0.2 pg/mL to 100 ng/mL, while the colorimetric mode provided visual semi-quantification with an LOD of 18.6 pg/mL across a linear range of 50 pg/mL to 100 ng/mL. This dual-mode configuration demonstrated significantly enhanced resistance to complex matrix interference compared to single-mode counterparts. In addition, to advance electrochemical multiplexing, Rahmanian et al. (75) constructed a dual-functional Fe_3_O_4_@AuNPs/ZIF-8 platform integrating electrochemical and fluorescent modalities. AuNPs facilitated dual signal transduction. In fluorescence mode, FAM-labeled aptamers immobilized on the surface underwent fluorescence quenching via FRET to proximal AuNPs, target binding induced aptamer displacement, disrupting FRET and restoring fluorescence proportional to AFB1 concentration with an LOD of 0.20 fg/mL. Meanwhile, in electrochemical mode, conformational changes of aptamers upon AFB1 binding impeded electron transfer at an AuNP-modified electrode, reducing redox current with an LOD of 0.32 pg/mL. This strategy achieved remarkable sensitivity and reliability across diverse complex matrices. A significant breakthrough by Liu et al. (76) integrated electrochemical and photoelectrochemical (PEC) modalities at a single interface using programmed light illumination synchronized with linear sweep voltammetry (LSV). Methylene blue (MB) intercalated within an AFB1 aptamer-cDNA duplex served as a bifunctional probe. AFB1 binding destroyed the duplex, releasing MB and decreasing both its redox current and photocurrent under illumination. The stable redox current of solution-phase [Fe(CN)_6_]^3−/4−^ acted as an internal reference. Simultaneous recording during a single LSV scan generated dual ratiometric readouts, yielding wide linear ranges and low LODs of 2.5 pg/mL and 4.7 pg/mL, respectively. Besides, SERS-based multiplexing reached new levels with the integration of magnetic relaxation switching (MRS) for simultaneous detection of AFB1, AFB2, and AFM1 (77). Label-free SERS tags (Au-Ag Janus NPs for AFB1, Au-mushroom NPs for AFB2) exhibited distinct intrinsic Raman peaks. Fe_3_O_4_@Au NPs functionalized with AFM1 aptamers served as MRS nanoprobes. Assembly via DNA hybridization created a single structure with strong SERS activity and high transverse relaxation time (T_2_). Target binding triggered dissociation of SERS tags (decreasing SERS signal for AFB1/AFB2) or dispersion of magnetic probes (reducing T_2_ for AFM1), enabling simultaneous, interference-free quantification with impressive LODs of 3.45 pg/mL for AFB1, 0.31 pg/mL for AFB2, and 0.42 pg/mL for AFM1, respectively.

**Figure 4:**
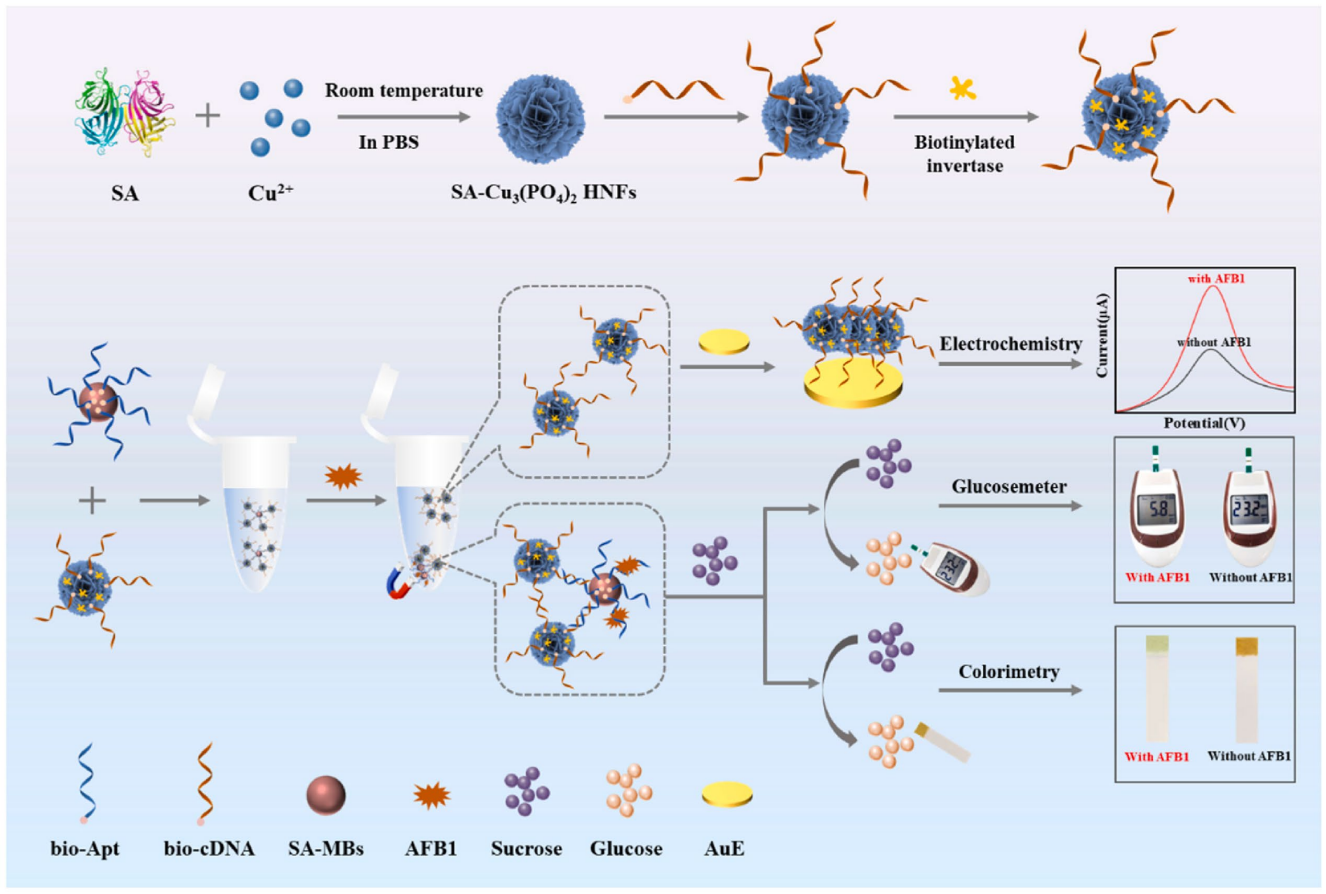
Schematic design of multi-mode biosensor for quantitative and precise detection of AFB1 via the integration of electrochemistry–glucosemeter–smartphone. Reproduced from Suo et al. (79) with permission from Elsevier.

Despite these established platforms, emerging frontiers emphasize deployable intelligence via innovative signal translation mechanisms and AI-enhanced data processing. Tang et al. (78) developed an enzyme-free colorimetric and photothermal dual-mode aptasensor for detection of OTA in corn. Their platform exploited a redox cycling amplification (RCA) system mediated by G-quadruplex-hemin/iodide complexes. These peroxidase-mimicking DNAzymes catalyzed TMB oxidation, producing both a visible color change detectable at 1 pg/mL and a measurable temperature increase under near-infrared irradiation with photothermal responses at 0.8 pg/mL. Integration with a common thermometer enabled instrument-free quantification, while smartphone RGB analysis simplified field deployment. Similarly, as shown in Figure 4, Suo et al. (79) created a portable multi-mode platform for AFB1 using streptavidin-functionalized copper phosphate hybrid nanoflowers (SA-Cu_3_(PO_4_)_2_ HNFs) conjugated with biotinylated invertase and cDNA. Magnetic separation captured target complexes, releasing SA-HNF probes for electrochemical detection with an LOD of 0.49 pg/mL. Crucially, invertase on captured probes converted sucrose to glucose, enabling rapid quantification via a portable glucometer sensitive to 5.4 pg/mL and colorimetric readout using urine glucose test strips sensitive to 3.7 pg/mL, processed by a custom smartphone app for RGB analysis, offering cross-verified field deployment. The most transformative development integrates machine learning with nanomaterial properties. Liu et al. (80) engineered Ti_3_C_2_ nanosheets functionalized with FAM-labeled ssDNA aptamers. The Ti_3_C_2_ substrate quenched FAM fluorescence and exhibited enhanced peroxidase-mimicking activity upon aptamer binding. Target recognition displaced ssDNA-FAM, restoring fluorescence detectable at 2.16 pg/mL and reducing catalytic TMB oxidation detectable at 1.58 pg/mL. A self-developed smartphone app captured images under controlled light, and processed extracted RGB values through a dual-channel fully connected artificial neural network. This machine learning model achieved highly accurate OTA prediction, establishing an intelligent sensing mode that transforms simple image data into quantitative toxin levels.

This evolution from fundamental optical/electrochemical hybrids to AI-driven platforms reveals a clear trajectory wherein dual-mode systems achieve synergistic signal integration rather than mere signal combination. Sensitivity remains paramount in optical designs, electrochemical hybrids dominate matrix complexity challenges, while enzyme-free amplification and portable readouts redefine field deployment. The convergence of orthogonal validation, nanomaterial engineering, and computational analytics establishes a new standard for reliable, real-world food safety monitoring.

## 4 Conclusion

Biosensors represent a rapidly evolving technological platform for mycotoxin detection, employing diverse biorecognition elements and transduction mechanisms to achieve targeted analyte quantification. Compared to conventional analytical methods such as ELISA and chromatographic techniques, biosensors demonstrate significant advantages in operational efficiency (rapid response, reduced assay time), analytical performance (high sensitivity and specificity), and practical utility (minimal reagent consumption, low operational skill requirements, and cost-effectiveness). Nevertheless, the early development of biosensors presents persistent challenges: critical knowledge gaps remain in detection universality, while inherent material limitations constrain real-world implementation. Notably, the instability of biological recognition components compromises measurement reproducibility and shelf-life, representing a fundamental barrier to commercial deployment. Addressing these limitations through biomolecular engineering and stabilization strategies constitutes a pivotal research frontier for advancing next-generation detection platforms.
